# Climate but Not Land Use Influences Body Size of Fowler's Toad (*Anaxyrus fowleri*)

**DOI:** 10.1002/ece3.71024

**Published:** 2025-02-27

**Authors:** Paradyse E. Blackwood, Amanda K. Martin, Jennifer A. Sheridan

**Affiliations:** ^1^ Department of Biological Sciences Purdue University West Lafayette Indiana USA; ^2^ Department of Zoology University of British Columbia Vancouver British Columbia Canada; ^3^ Section of Amphibians and Reptiles Carnegie Museum of Natural History Pittsburgh Pennsylvania USA

**Keywords:** *Anaxyrus fowleri*, climate change, forest area, mass, museum collections, snout–vent length

## Abstract

Anthropogenic changes to the environment have been associated with changes in body size of multiple organisms. However, although climate and land use influences on body size have been examined separately, the simultaneous effects and potential interactions of these two factors on body size have rarely been studied. We examined the size and mass of a common North American toad (Fowler's toad, *Anaxyrus fowleri* ) using museum specimens from 1931 to 1998 to quantify the potential interactive effects of climate change (temperature and precipitation) and land use change (forested area) on body size. We found that snout–vent length (SVL) and mass declined over time, and that size was negatively related to both temperature and precipitation (smaller size at higher values of temperature and precipitation). We did not find evidence of an effect of forest cover on size or mass. Our results suggest that Fowler's toad body size is affected by climate but not land use, and we encourage further examination of additional species and land cover variables (such as urbanization) to determine whether our results are representative of ectotherms more broadly. This work highlights the strength of climate in determining anuran body size and contrasts with existing studies showing interactive effects of climate and land use on animal body size.

## Graphical abstract

We found that snout–vent length (SVL) and mass of Fowler's toads ( *Anaxyrus fowleri* ) declined over time, and that size was inversely related to temperature and precipitation. We found no effect of forest cover on size. This suggests that observed increases in temperature and precipitation have significantly contributed to size declines in this species, but land use does not significantly affect the size of *A. fowleri* .

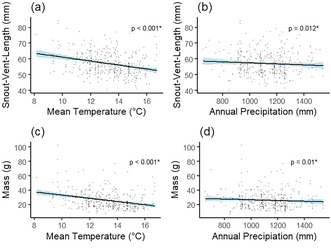

## 1 Introduction

Body size is a critical trait in ecology that influences many facets of an organism's survival and reproduction (Calder 1984; Roff 2002). In particular, morphology affects physiological and ecological mechanisms, including thermoregulation (Weathers 1981; Berec et al. 2014), metabolism (Brown et al. 2004), mobility (Jenkins et al. 2007), energy utilization (Post and Parkinson 2001), and reproductive output, such as clutch size (Chamaillé‐Jammes et al. 2006; Van Gils et al. 2016), all of which contribute to lifetime fitness. Thus, widespread changes in body size have the potential to impact population persistence, community interactions, and species survival.

Recently, studies have demonstrated widespread changes in size across an array of taxa with respect to both climate and land use change (Gardner et al. 2019; Kelemen and Rehan 2021; Sheridan, Martin, et al. 2022; Wood and Cousins 2023), two of the greatest threats to species persistence. In endotherms, larger body size occurs in colder environments at high latitudes to decrease the surface area to volume ratio for efficient heat conservation (Bergmann 1847; Zamora‐Camacho et al. 2014; Alston et al. 2023). In ectotherms, increasing temperatures can affect morphology and phenology directly through the influence on metabolism and development rate (Atkinson 1994; Gillooly et al. 2001) such that as temperatures warm, the size of organisms declines (e.g., temperature‐size rule; Gardner et al. 2011; Sheridan and Bickford 2011; Ohlberger 2013). Although ectotherms have been shown to be smaller at higher temperatures in experimental and theoretical settings (Atkinson 1994; Gillooly et al. 2001), broad geographic trends of body size show that some follow the pattern of larger sizes at cooler temps, whereas others show no trend or the opposite (Ashton and Feldman 2003; Laugen et al. 2005; Adams and Church 2008; Amado et al. 2019). However, because size declines are not uniform across species (Gardner et al. 2011; Sheridan and Bickford 2011; Guralnick et al. 2020), size changes associated with climate warming can lead to changes in ecological communities.

In addition to temperature, other climate and land use variables can influence body size. Precipitation, for example, can influence the distribution, body size, and physiology of organisms (Yom‐Tov and Geffen 2006; Morales‐Castilla et al. 2012; Kelly et al. 2018). Precipitation can lead to increased size when it leads to increased resource availability through its influence on primary productivity (Rosenzweig 1968; Blois et al. 2008; O'Keefe et al. 2013) or lead to decreased size when organisms must prioritize desiccation resistance (Nevo 1973; Olalla‐Tárraga et al. 2009; Pincheira‐Donoso et al. 2019). Habitat fragmentation can lead to larger body size as organisms are required to travel greater distances to access suitable resources (Fietz and Weis‐Dootz 2012; Hillaert, Hovestadt et al. 2018; Hillaert, Vandegehuchte, et al. 2018). Fragmentation and urbanization can reduce the connectivity of ecological resources, thus favoring individuals with larger body size and greater mobility (Piano et al. 2017; Merckx and Van Dyck 2019) and generalist species in urban areas may be able to exploit abundant anthropogenic resources, resulting in larger body size (Auman et al. 2008; Jessop et al. 2012). Conversely, fragmentation and urbanization can lead to higher ambient temperatures (Oke 1982; Brans et al. 2017; Merckx et al. 2018), contributing to size declines. In the coming decades, land use change and urbanization are expected to increase (Tilman et al. 2017), and although there is a sizeable literature exploring how land use and climate change separately influence body size, very few studies have examined their combined impact on body size.

To better predict organism size under future climate and land use scenarios, it is necessary to examine potential interactions between them. Though relatively rarely reported on, studies have shown that as temperatures increase, the effect of precipitation on size changes, indicating an interaction between temperature and precipitation on size that is not often examined (Martínez‐Monzón et al. 2018; Davy et al. 2022; Sheridan, Mendenhall, et al. 2022). Conversely, because there are so few studies that explicitly test the interaction between climate and land use on body size, it is difficult to determine whether common trends exist. For example, there is evidence for stronger mammalian size declines with rising temperatures in urban versus rural areas (Hantak et al. 2021), but there are no significant interactions between temperature and urbanization for bird bill size (Miller et al. 2018) or between temperature and forest cover for bat wing size (Wood and Cousins 2023). The cited examples focus on endotherms, with no studies to our knowledge examining the combined effects of climate and land use changes on ectotherms, which should be more sensitive to such changes than endotherms. Without further evidence, it is difficult to determine whether the combination of land use and climate change is likely to have significant nonadditive effects on body size of both endotherms and ectotherms.

To better understand how the combination of climate change (temperature and precipitation) and land use (forested area) influences body size, we examined snout–vent length (SVL) and mass of a common North American amphibian ( *Anaxyrus fowleri* ) from 1930 to 2020. Amphibians are a good taxon to explore the interactive effects of land use and climate on body size because they are highly sensitive to fine‐scale changes in temperature and precipitation (Blaustein et al. 2010; Sheridan et al. 2018; Pincheira‐Donoso et al. 2019; Guo et al. 2019) and alterations of habitat or land use (Blaustein et al. 2011; Rohr and Palmer 2013; Reid et al. 2019). Several amphibian species have shown changes in body size in response to climate change or land use (Reading 2007; Caruso et al. 2014; Murphy et al. 2016; Martínez‐Monzón et al. 2018; Jennette et al. 2019; Sheridan, Mendenhall, et al. 2022), with multiple studies showing declines with climate warming or with increased urbanization. These trends are not universal, however, and the present study will be the first to our knowledge to examine potential interactions between climate and land use on amphibian body size. Based on prior studies, we predicted that the body size of *A. fowleri* (an ectotherm with limited dispersal ability) would decrease over our study period, with an interaction between temperature and precipitation affecting body size. Furthermore, we expected individuals inhabiting areas with less forest cover would be smallest because lower forest cover is associated with decreased food resources and is expected to exacerbate warming.

## 2 Materials and Methods

### 2.1 Study System

We examined Fowler's toad ( *A. fowleri* ; Family: Bufonidae), which is plentiful in museum collections from a wide range of years. The native range of *A. fowleri* includes most of the eastern USA where they are considered abundant (Babbitt 1937; Wright and Wright 1949; Martof 1980; Klemens 1993; Powell et al. 2016) and are typically found in temperate forests, suburban and urban areas, and river valleys (Wright and Wright 1949; Powell et al. 2016). Fowler's toads have rapid development and a short larval period (40–60 days) prior to emerging as metamorphs, and environmental conditions, such as climate change have been shown to influence growth and emergence within the larval stage (Wright and Wright 1949; Green et al. 2016; Green 2017). Thus, Fowler's toads are very likely to be impacted by both climate and land use variables, such as temperature, precipitation, and/or forest cover. Although *A. fowleri* may hybridize with other toads, such as *A. americanus* (Green 1996; Green and Parent 2003), many museums label hybrids (e.g., *Anaxyrus americanus* × *Anaxyrus fowleri* ). We acknowledge that hybrids may still occur under the label *A. fowleri* , but trust that museums make every effort to identify likely hybrids, and trained herpetological collections managers can decipher *A. fowleri* from *A. americanus* .

### 2.2 Specimen Measurement, Climate, and Land Use Data

**FIGURE 1:**
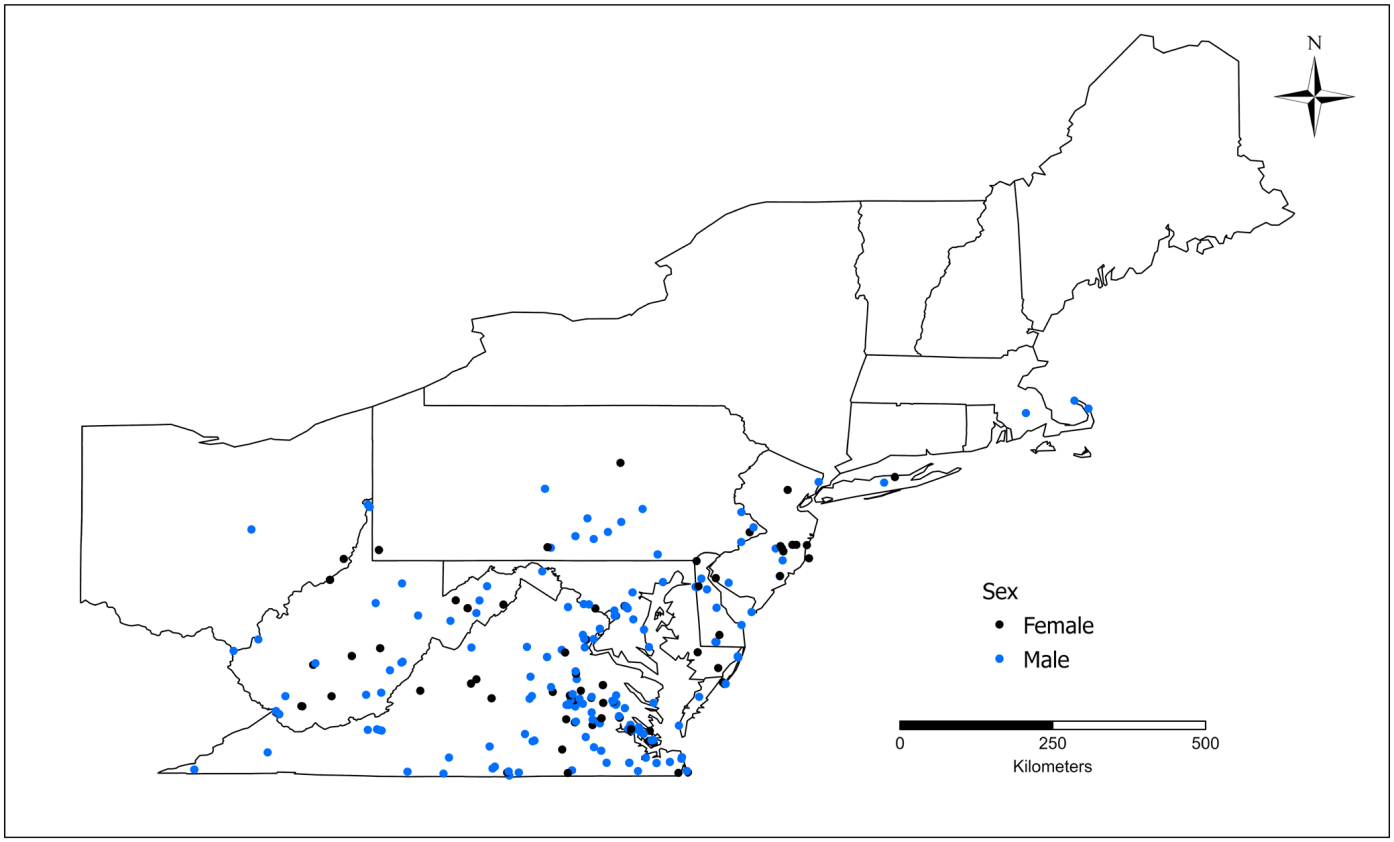
Collection localities of *Anaxyrus fowleri* specimens from the northeastern United States used in the data analysis for this study.

We examined sexually mature specimens from the Carnegie Museum of Natural History (CM; Pittsburgh, PA, USA), American Museum of Natural History (AMNH; New York, NY, USA), the Museum of Comparative Zoology at Harvard (MCZ; Boston, MA, USA), The National Museum of Natural History (NMNH; Washington, D.C, USA), and the North Carolina Museum of Natural Sciences (NCSM; Raleigh, NC, USA). We included in our dataset only individuals with data on latitude, longitude, and year of collection. We measured sexually mature *A. fowleri* from the northeastern United States collected between 1930–2020 (Figure 1). All specimens were measured by one of two individuals (J.A.S. and P.E.B.) to minimize inter‐individual measurement error. Each specimen was measured three times, and the average of those measurements was used in statistical analyses (i.e., mean SVL). Male sexual maturity was determined by the presence of secondary sexual characteristics (black/dark throat and nuptial pads; Powell et al. 2016). Female sexual maturity was determined by the presence of eggs (gravid) or enlarged oviducts for individuals with SVL > 45 mm (Wright and Wright 1949; Martof 1980; Kellner and Green 1995). We used 45 mm as a cutoff to exclude juveniles of each sex, in line with previous work on this species (Green and Parent 2003) and given that additional studies list the minimum adult size as 50 mm (Wright and Wright 1949; Powell et al. 2016). For SVL measurements, specimens were gently flattened and straightened, and measured three times to the nearest 0.05 mm with a digital caliper, and the mean of these values was used for analyses. For mass measurements, each specimen was removed from ethanol and gently patted with a paper towel twice on each surface (dorsal and ventral), then placed on a digital scale and weighed to the nearest 0.5 g.

Because of uneven availability of specimens across latitude and decade, we trimmed our dataset in the following ways: (1) any given location (latitude and longitude) with large numbers of individuals collected in a single year was randomly subsampled to include a maximum of 20 individuals from that location and year in the final dataset; (2) we examined sample sizes of each decade from each degree of latitude and used a random number generator to randomly subsample our data to produce roughly even numbers of samples across decades and eliminate any significant relationship between latitude and year. Thus, from nearly 1000 total measured individuals, our final dataset included 394 individuals (139 females and 255 males) collected between 1931 and 1998 (Table S1).

For each specimen's locality, we extracted temporally specific climate data from Parameter‐elevation Regressions on Independent Slopes Model (PRISM; 2023, PRISM Climate Group, Oregon State University) in ArcGIS Pro (Esri, Redlands, California, USA). Using PRISM, we obtained mean annual temperature (°C) and total annual precipitation (mm) for each specimen based on its collection location and year, with 30 arc‐second resolution.

We extracted the percentage of forest area within a 1 km buffer for each specimen derived from the United States Geologic Survey land cover dataset (USGS; 1938–1992; Sohl et al. 2016), and National Land Cover Database (NLCD 2001; https://www.mrlc.gov/data). We chose 1 km because *A. fowleri* are generally sedentary and most individuals move about 500 m or less in a season (Clark 1974). For individuals collected from 1931 to 1937, we used land cover data from 1938 because digital land use maps are unavailable prior to 1938. We selected 1931 as the earliest date of sampling to take advantage of large numbers of specimens collected in the 1930s and because the 1938 land cover data is likely a reasonable estimate for land cover during this time period. For individuals collected from 1994 to 1995, we used land cover data from 1992; for individuals collected in 1998, we used land cover data from 2001, as these were the nearest years with land cover data. Before data extraction, we reclassified the USGS and NLCD datasets to combine all “forest” categories (deciduous forest, evergreen forest, mixed forest) into a single “forest” variable. We then resampled each land cover dataset (spatial resolution: USGS 250 m, NLCD 30 m) to 300 m spatial resolution. Within each 1 km buffer, we calculated the proportion of forest area on the basis of latitude, longitude, and collection year.

### 2.3 Statistical Analysis

All data were analyzed in R (version 4.3.1; R Core Team 2023). All variables were transformed using the *scale* function in R, which standardize the data by subtracting the mean and dividing by the standard deviation. Because several papers examine change in length and mass over time without directly analyzing those changes related to climate or land use, we examined change in SVL and mass over time using linear regressions to enable comparison of our results to previous studies (Figures S1 and S2). We also examined changes in temperature, precipitation, and percent forest area over time (Figures S3–S5).

We examined the relationship between SVL and mass (separately) and the explanatory variables latitude, temperature, precipitation, and percent forest area using linear mixed models. We included sex as a random factor because we know a priori that females and males differ in size, and our goal was not to quantify how sex predicts SVL or mass. Because latitude and temperature are strongly correlated, they could not be included in the same model, so for both SVL and mass, we ran two sets of global models: one with temperature and one with latitude, together with the other variables and all possible two‐way interactions of those terms. The global models were followed by backward stepwise selection to find the best‐fit model using *lmerTest* (Kuznetsova et al. 2017). Top models were compared using AIC to get one final top model each for SVL and mass. We obtained marginal and conditional *R* ^2^ values for each best‐fit model using the *MuMIn* package (Bartoń 2023). We confirmed that our data met the assumptions of linear mixed models (package: *performance*, function: *check_collinearity*, *check_model*; Lüdecke et al. 2021). We also checked model variance inflation factors of predictors to guarantee that VIFs were below 2 and that there was no multicollinearity (package: *car*; function: *vif*; Fox (2019)). All data and code needed to reproduce statistical tests and plots are included as Supporting Information.

## 3 Results

### 3.1 Size, Temperature, Precipitation, and Forest Cover Changes Over Time

Snout–vent length of both male and female *A. fowleri* significantly decreased over time (*F*_1,252_ = 10.69; *p* < 0.001 and *F*_1,138_ = 7.41; *p* = 0.007, respectively; Table S2, Figure S1), as did mass (*F*_1,252_ = 14.33; *p* < 0.001 and *F*_1,138_ = 7.47; *p* = 0.007, respectively; Table S2, Figure S2). Mean annual temperature (*F*_1,392_ = 17.8; *p* < 0.001; Table S2, Figure S3) and total annual precipitation (*F*_1,392_ = 16.02; *p* < 0.001; Table S2, Figure S4) significantly increased from 1931 to 1998. We found no change in percent forest area (*F*_1,392_ = 1.82; *p* = 0.178; Table S2, Figure S5) from 1931 to 1998.

### 3.2 Snout–Vent Length

**FIGURE 2:**
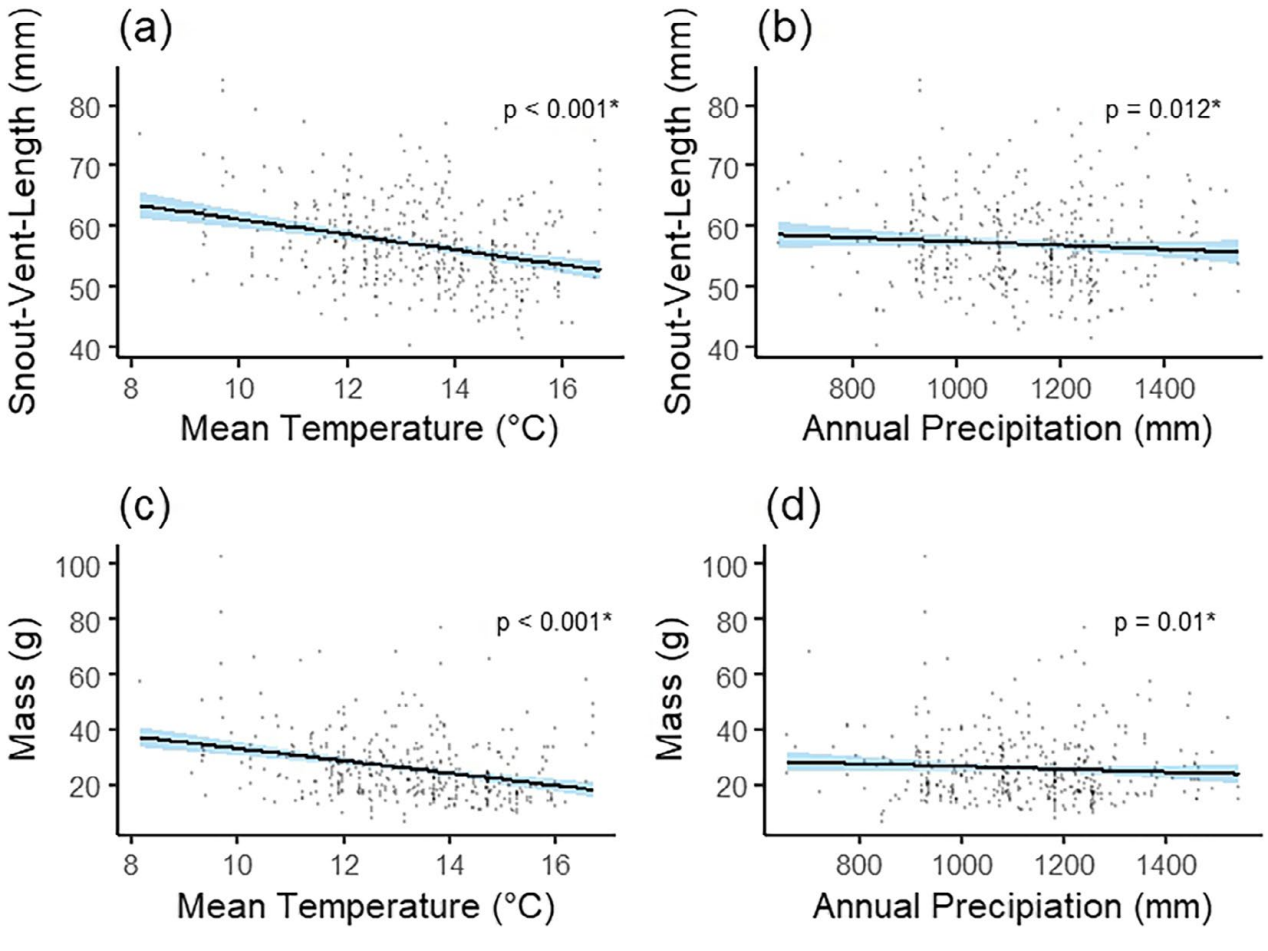
*Anaxyrus fowleri* snout–vent length related to (a) annual mean temperature (°C) and (b) annual precipitation (mm). *Anaxyrus fowleri* mass related to (c) annual mean temperature (°C) and (d) annual mean precipitation (mm). Shading around the line represents a 95% confidence interval.

The best model of *A. fowleri* SVL included mean annual temperature (*p* < 0.001, Figure 2a) and total annual precipitation (*p* = 0.01; Table 1; Figure 2b; conditional *R* ^2^ = 0.461, marginal *R* ^2^ = 0.071), indicating that size is negatively related to both temperature and precipitation. These data suggest that the observed decreases in *A. fowleri* SVL over the study period (above) are due to concurrent increases in temperature and precipitation over the same time period (above).

**TABLE 1:** Estimates of fixed effects from the linear mixed model quantifying the snout–vent length (SVL) of *Anaxyrus fowleri* from 1931 to 1998. Model structure: SVL *~* Temperature + Precipitation + (1 |sex). Mean annual temperature and total annual precipitation were scaled prior to analyses to meet linear modeling assumptions. SE is the standard error and df are the degrees of freedom. Significant *p* values are bolded.

Term	Estimate	SE	df	*t*	*p*
Intercept	58.12	3.56	1.00	16.31	**0.039**
Temperature	−1.97	0.30	390.0	−6.61	**< 0.001**
Precipitation	−0.79	0.30	390.0	−2.63	**0.009**

### 3.3 Mass

The best‐fit model of *A. fowleri* mass included mean annual temperature (*p* < 0.001; Figure 2c) and total annual precipitation (*p * = 0.01; Table 2; Figure 2d; conditional *R* ^2^ = 0.498, marginal *R* ^2^ = 0.070). These data suggest that the observed declines in *A. fowleri* mass over time are due to the concurrent increases in temperature and precipitation observed in our study area over the study period (above).

**TABLE 2:** Estimates of fixed effects from the linear mixed model quantifying the mass of *Anaxyrus fowleri* from 1931 to 1998. Model structure: Mass ~ Temperature + Precipitation + (1 |sex). Mean annual temperature and total annual precipitation were scaled prior to analyses to meet linear modeling assumptions. SE is standard error and df are degrees of freedom. Significant *p* values are bolded.

Term	Estimate	SE	df	*t*	*p*
Intercept	27.87	6.58	1.0	4.23	0.148
Temperature	−3.48	0.51	390.0	−6.87	**< 0.001**
Precipitation	−1.31	0.51	390.0	−2.58	**0.010**

## 4 Discussion

We found that *A. fowleri* body size decreased from 1931 to 1998, with size negatively related to temperature and precipitation. Specifically, we found that size was smaller at higher temperatures and values of precipitation, similar to previous studies (Caruso et al. 2014; Horne et al. 2017; Tseng et al. 2018; Sheridan, Mendenhall, et al. 2022). As temperatures warm, ectotherm metabolism increases (Gillooly et al. 2001; Dillon et al. 2010), so without increased resources to compensate for increased metabolism, organisms are expected to shrink as global warming continues. Previous studies have found increased precipitation associated with size declines in some beetles (Tseng et al. 2018; Baar et al. 2018) and birds (Weeks et al. 2020; Onley et al. 2020). In these studies, however, the effect of precipitation was not uniform across species, and few details have been offered to explain the inverse relationship between precipitation and size. Theoretical work indicates that precipitation can contribute positively to body size via an increase in primary productivity (Rosenzweig 1968) or negatively through a selection for desiccation resistance (Bujan et al. 2016). In the latter case, which is likely relevant to *A. fowleri* , organisms are larger at low levels of precipitation because large bodies have a lower surface area: volume ratio and are therefore less prone to desiccation. Thus, there is selection for larger size at low levels of precipitation, which is relaxed at higher values of precipitation.

We did not find evidence of an effect of forest cover on size or mass. Although there have been a handful of studies testing for interactions between climate and land use on body size of endotherms (reviewed by Martin and Sheridan 2022), few consistent results have emerged. For example, Hantak et al. (2021) examined more than 100 mammal species and found that temperature and urbanization interact to predict body size, with greater mass declines in high versus low human density areas as temperature increases. Wood and Cousins (2023) examined cranial size and wing morphology of three species of bats with respect to temperature and forest cover. Although they found that both temperature and forest cover affected wing shape, forearm length (a measure of body size) was not related to temperature for any species and had a significant (negative) relationship with forest cover for only one species. Thus, although there is some evidence that urbanization or human population density may affect size and interact with climate changes to affect size (Miller et al. 2018; Hantak et al. 2021), body size responses to forest cover have mixed support. Our data suggest that *A. fowleri* body size is affected by climate (temperature and precipitation) but not land use (forest cover), but additional studies on ectotherm species are needed to determine whether our results are representative of ectotherms more broadly. Although many ectotherm studies have focused on how individual climate or land use variables (i.e., temperature only, interaction of temperature and precipitation) have impacted ectotherm body size (Desai and Singh 2009; Nowicki et al. 2012; Sheridan et al. 2018; Pincheira‐Donoso et al. 2019; Guo et al. 2019), ours is the first to our knowledge to explicitly test for potential interactions between climate and land use variables on ectotherm body size. We encourage additional examination of interactions between climate and land use, including other types of land cover, on organism size responses, especially for ectotherms which often receive less attention than endotherms despite representing the majority of life on earth.

Ectotherms, especially amphibians and reptiles, may receive less attention on this topic due in part to some studies indicating that geographic variation in size is not strongly predicted by temperature, for example (Ashton and Feldman 2003; Olalla‐Tárraga et al. 2006; Slavenko and Meiri 2015; Henry et al. 2023). However, recent work has indeed demonstrated that recent climate warming is associated with size declines of amphibians and reptiles (Reading 2007; López‐Calderón et al. 2017; Sheridan, Martin, et al. 2022; Sheridan, Mendenhall, et al. 2022), but not universally so (Chamaillé‐Jammes et al. 2006; Stanley et al. 2020). Furthermore, there is some evidence that water availability is an important predictor of amphibian body size (Gouveia and Correia 2016; Sheridan, Mendenhall, et al. 2022) which may be overlooked in studies examining geographic or temporal variation in body size variation. We encourage further examination of responses of additional ectotherms with respect to both temperature and precipitation, particularly amphibians.

With observed changes in the morphology of natural populations, scientists often discuss whether such changes are genetic or plastic to better understand how organisms adapt to novel environments. It is important to understand the mechanisms that underlie the responses to predict how organisms will keep up with anthropogenic changes to the environment (Diamond and Martin 2016). In this study, our goal was to determine whether there have been any significant body size changes associated with climatic and land use alterations, meaning we did not explicitly test whether the changes in body size were a plastic and/or evolutionary response. The body size reductions of Fowler's toad may relate to the temperature‐size rule which describes the phenotypically plastic response of body size to temperature where individuals reared in warmer temperatures are smaller (Atkinson 1996; Angilletta Jr et al. 2004). Additionally, our data demonstrate that Fowler's toad size is negatively related to precipitation, in line with the desiccation resistance hypothesis. Although there is some suggestion that desiccation resistance may be an evolutionary response across species, with desiccation‐resistant species inhabiting drier environments (Bujan et al. 2016), within‐species variation in desiccation resistance may be plastic (Nevo 1973). A literature review and meta‐analysis examining evidence for evolutionary and plastic responses to climate change in anurans determined that most studies found phenotypically plastic responses; however, there were fewer studies documenting adaptive evolution, and the authors recommended more studies be conducted to examine genetic changes (Urban et al. 2014). Although fewer studies have investigated the types of responses amphibians have to land use changes, some studies have found that responses occurred because of phenotypic plasticity or a mix of both genetic evolution and plasticity (Bókony et al. 2021). Further research of this species is needed to determine whether this species' size responses are due to phenotypic plasticity and/or genetic evolution (Franks et al. 2014).

Our results indicate that temperature and precipitation affect amphibian body size. *Anaxyrus fowleri* SVL and mass have significantly decreased over time, as temperature and precipitation have increased. Body size declines can influence reproductive output, thermoregulation, and mobility (Roff 2002; Jenkins et al. 2007; Gardner et al. 2011; Brown et al. 2017), ultimately leading to increased thermal stress and population declines (Roff 2002; Buckley 2021; Peralta‐Maraver and Rezende 2021). Additionally, amphibian body size impacts fitness and survivorship, so body size declines can be detrimental for populations (Yagi and Green 2016; Székely et al. 2020). Increased energetic demands, altered developmental and growth rates, and higher disease susceptibility are associated with changes in amphibian thermoregulation and mobility (Carey et al. 2006; Blaustein et al. 2010, 2011; Bickford et al. 2010; Wu et al. 2018), which in turn can decrease survival. Experimental studies and further research are needed to determine the underlying mechanisms affecting amphibian body size under altered climate and land use scenarios. Future work should examine other ectotherm species to determine how climate and land use variables, and their potential interactions, affect morphology as the environment changes. Understanding the individual and combined effects of land use and climate on body size will aid in predicting population and community dynamics with ongoing anthropogenic change.

## Author Contributions

**Paradyse E. Blackwood:** conceptualization (equal), data curation (equal), formal analysis (lead), funding acquisition (lead), investigation (lead), methodology (equal), project administration (equal), writing – original draft (lead), writing – review and editing (equal). **Amanda K. Martin:** conceptualization (equal), data curation (equal), formal analysis (equal), investigation (equal), methodology (equal), writing – review and editing (equal). **Jennifer A. Sheridan:** conceptualization (equal), data curation (equal), formal analysis (equal), investigation (equal), methodology (equal), project administration (equal), supervision (lead), writing – original draft (equal), writing – review and editing (equal).

## Conflicts of Interest

The authors declare no conflicts of interest.

## Supporting information


Data S1.

## Data Availability

Data are available as Supporting Information.
