# Supplementary material for: Climate but Not Land Use Influences Body Size of Fowler's Toad (Anaxyrus fowleri)
Source: Ecol Evol. 2025 Feb 27;15(3):e71024. doi: 10.1002/ece3.71024 (PMC11868702; doi:10.1002/ece3.71024)
Supplement: Supplementary file 1 — Data S1. [file ECE3-15-e71024-s001.zip › Blackwood et al. SupplInfo_CleanCopy_6Nov2024.docx]

Supplemental Information for “Climate but not land use influences body size of Fowler’s toad (*Anaxyrus fowleri*)”

Paradyse E. Blackwood^1^, Amanda K. Martin^2^, Jennifer A. Sheridan^2*^

*^1^Department of Biological Sciences, Purdue University, West Lafayette, IN USA 47907*

*^2^Section of Amphibians and Reptiles, Carnegie Museum of Natural History, Pittsburgh, PA USA 15213*

**Correspondence may be addressed to:*

*Jennifer A. Sheridan*

*Carnegie Museum of Natural History*

*4400 Forbes Avenue, Pittsburgh, Pennsylvania USA 15213*

[*jasheridan@gmail.com*](mailto:jasheridan@gmail.com) *(JAS)*

Table S1. Number of specimens per decade for each degree of latitude in the final dataset of the present study.

| **Latitude** | **1930s** | **1940s** | **1950s** | **1960s** | **1970s** | **1980s** | **1990s** |
| --- | --- | --- | --- | --- | --- | --- | --- |
| **36** | 0 | 9 | 4 | 3 | 10 | 10 | 0 |
| **37** | 26 | 17 | 41 | 10 | 25 | 25 | 29 |
| **38** | 25 | 10 | 6 | 2 | 24 | 4 | 1 |
| **39** | 15 | 15 | 12 | 7 | 19 | 5 | 2 |
| **40** | 10 | 5 | 5 | 6 | 1 | 1 | 10 |

Table S2. Estimated marginal means from changes over (1930–2020). SE is standard error, df is degrees of freedom. Significant p-values are in bold.

| **Variable** | **Estimate** | | **SE** | **F-stat_df_** | **p-value** |
| --- | --- | --- | --- | --- | --- |
| Male SVL | -0.055 | 0.017 | | F_1,252_ = 10.69 | **0.001** |
| Female SVL | -0.089 | 0.033 | | F_1,138_ = 7.41 | **0.007** |
| Male Mass | -0.086 | 0.023 | | F_1,252_ = 14.33 | **<0.001** |
| Female Mass | -0.177 | 0.065 | | F_1,138_ = 7.47 | **0.007** |
| Mean Annual Temperature | 0.017 | 0.004 | | F_1,392_ = 17.80 | **<0.001** |
| Mean Annual Precipitation | 1.641 | 0.410 | | F_1,392_ = 16.02 | **<0.001** |
| Percent Forest Cover | 0.001 | 0.001 | | F_1,392_ = 1.82 | 0.178 |


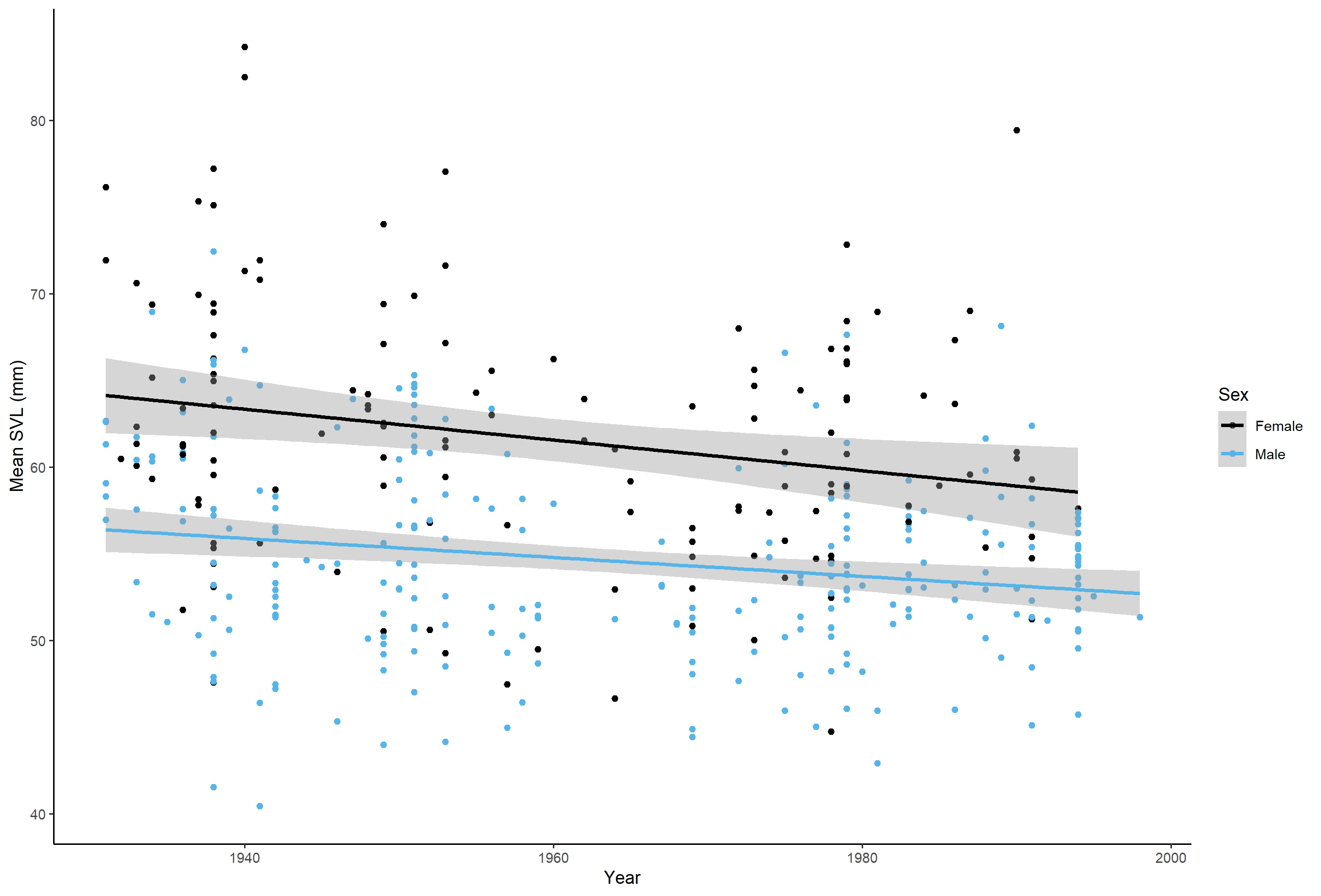


Figure S1. Change in *A. fowleri* SVL over time. Error bars represent 95% confidence intervals.


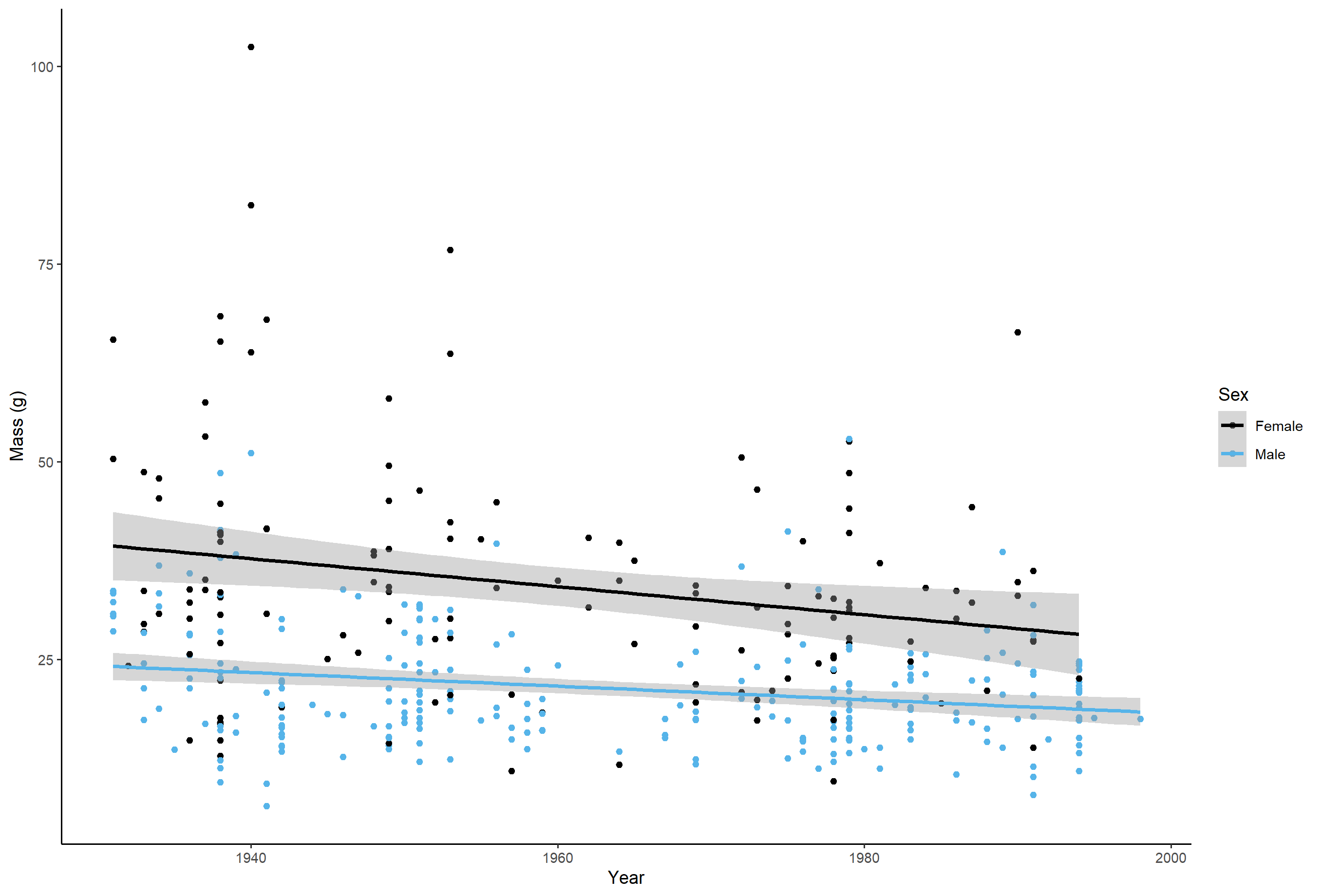


Figure S2. Change in *A. fowleri* mass over time. Error bars represent 95% confidence intervals.


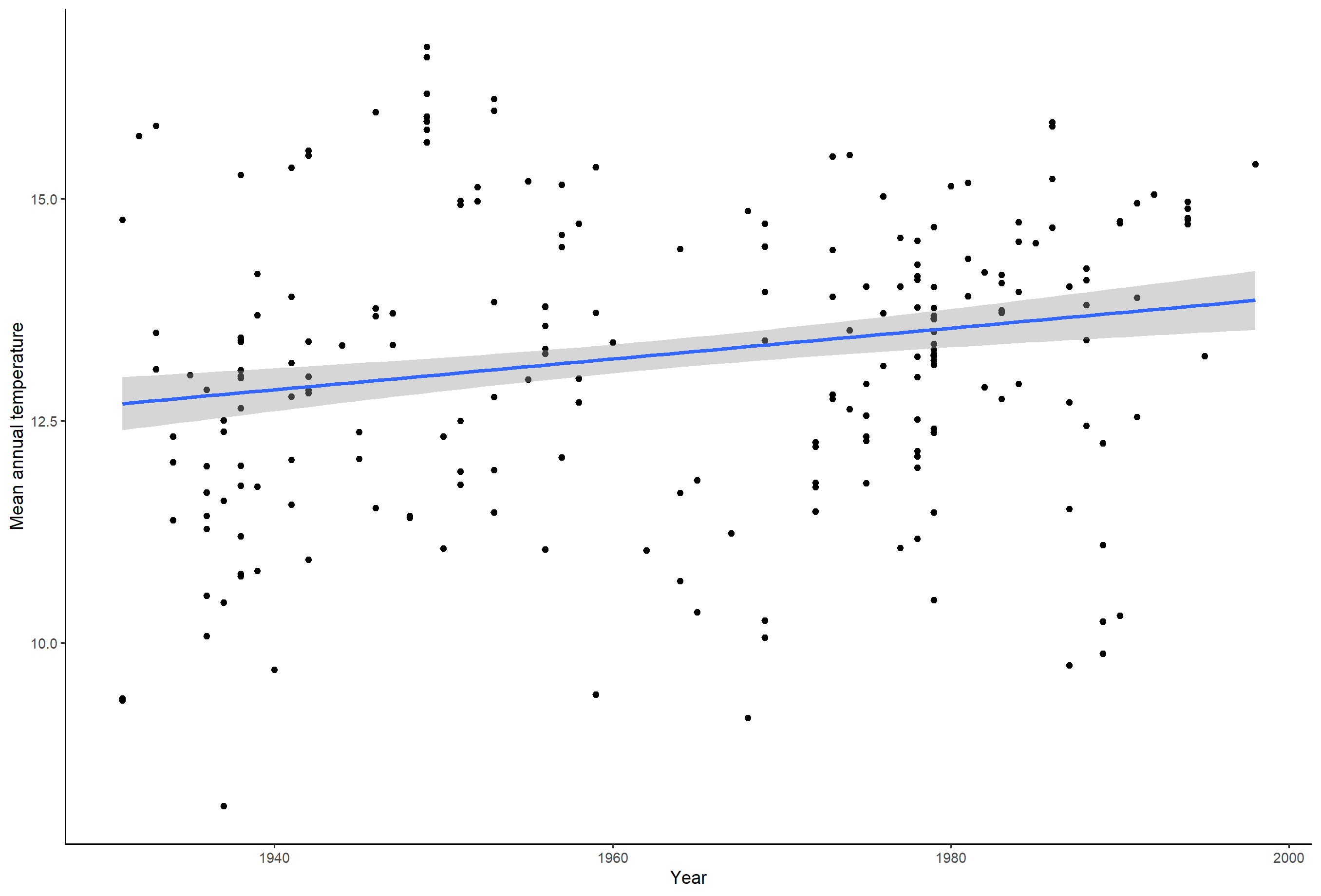


Figure S3. Change in mean annual temperature over time for the study area. Error bars represent 95% confidence intervals.


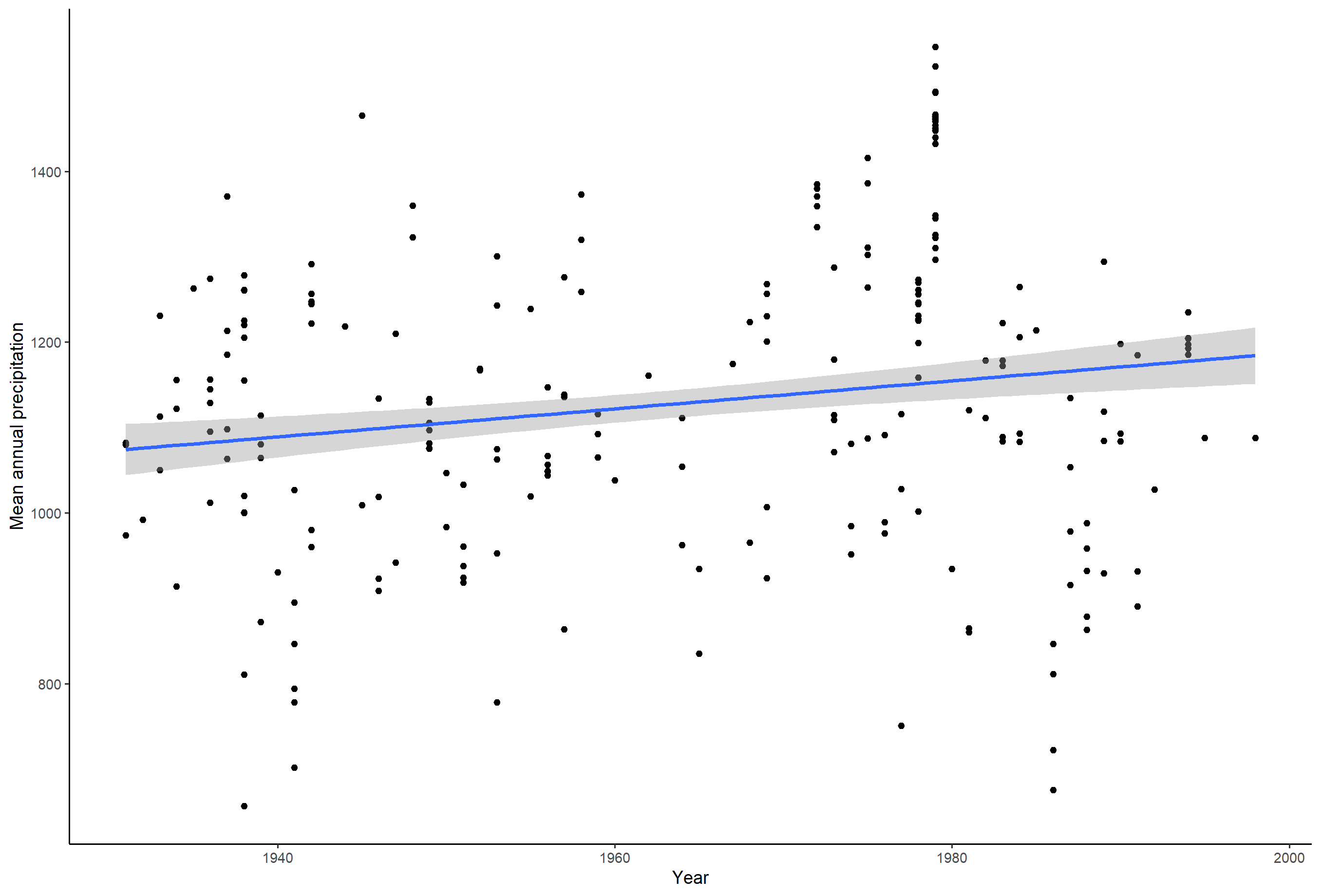


Figure S4. Change in mean annual precipitation over time for the study area. Error bars represent 95% confidence intervals


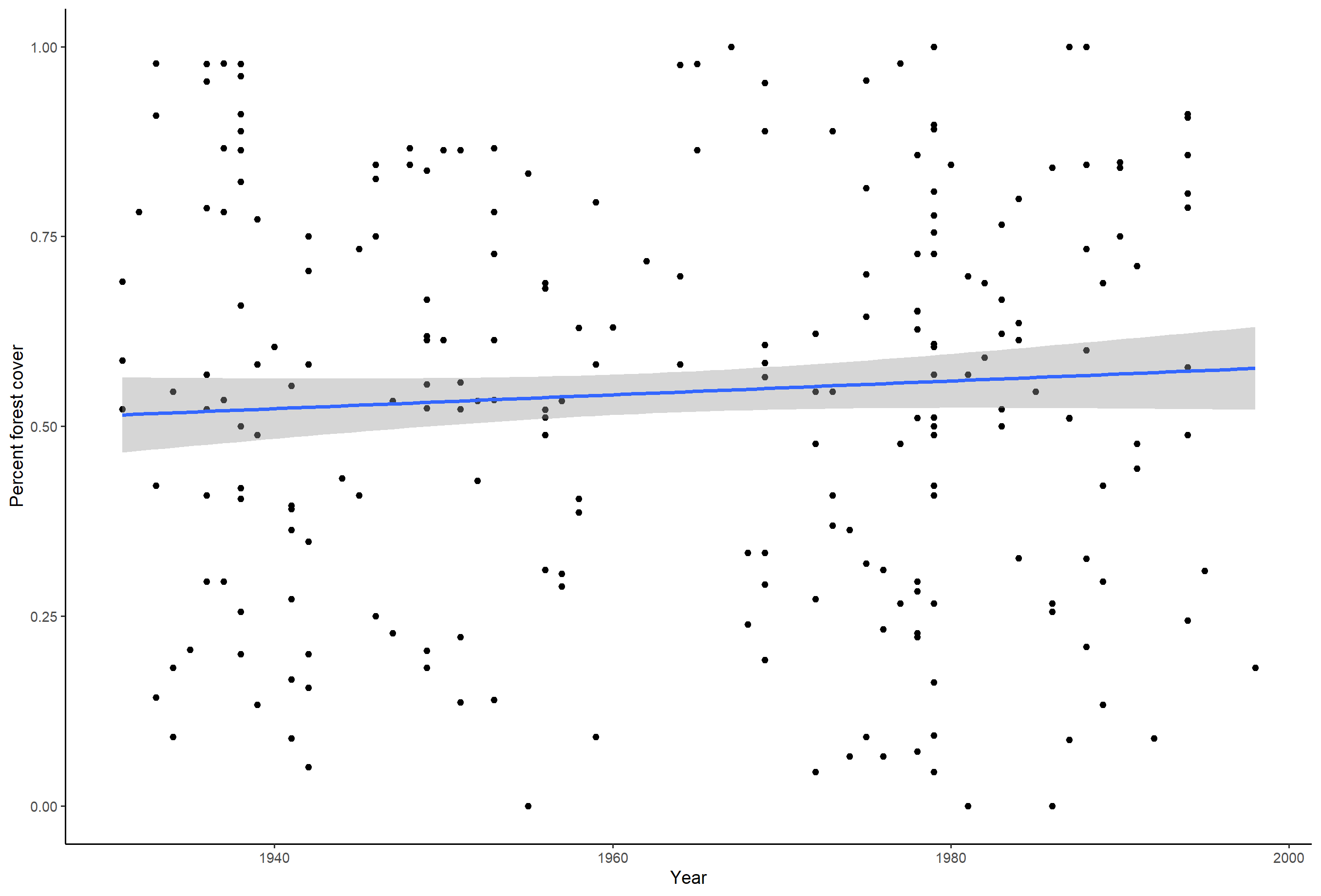


Figure S5. Percent forest cover over time for the study area. Error bars represent 95% confidence intervals.
